# Surgical diagnosis and treatment of a solitary lung nodule of IgG4-related disease, mimicking primary lung carcinoma or metastatic lung tumour: A rare case

**DOI:** 10.1016/j.ijscr.2023.108435

**Published:** 2023-06-22

**Authors:** Akira Haro, Tomoyuki Hida

**Affiliations:** a Department of Chest Surgery, Onga Nakama Medical Association Onga Hospital, Japan; b Department of Radiology, Onga Nakama Medical Association Onga Hospital, Japan

**Keywords:** IgG4-related lung disease, Primary lung cancer, Metastatic lung tumour, Radiological characteristics, Surgery

## Abstract

**Introduction:**

IgG4-related disease is a poorly understood immune disorder. Its features include tumour-like swelling of involved organs, lymphoplasmacytic infiltrate with IgG4 positive plasma cells. IgG4-related lung disease can manifest radiologically as various types of pulmonary abnormalities, including mass-like lesions and pleural effusion, and it may mimic malignant disease.

**Presentation of case:**

A 76-year-old man was found to have a 4-mm ground glass opacity in the left lower lobe of the lung on follow-up chest CT after surgery for colon carcinoma. This lesion gradually became consolidated and enlarged to 9 mm over about three years. We performed a video-assisted left basal segmentectomy for the purposes of both diagnosis and treatment. Pathological examination revealed lymphoplasmacytic infiltration, mainly with IgG4-positive plasma cells.

**Discussion:**

A major characteristic of IgG4-related lung disease is multiple, small, bilateral, lung nodules and solid nodules reportedly being detected in almost all patients. However, solitary nodules are rare, being present in only 14 %. Moreover, this case shows extremely rare radiological findings in which a ground-glass opacity had gradually morphed into a solid nodule. It is difficult to differentiate IgG4-related lung nodules from other lung diseases, such as primary or metastatic lung tumours, standard interstitial pneumonia, organizing pneumonia.

**Conclusion:**

We have here presented a rare case of IgG4-related lung disease with a 3-year course, including detailed radiological findings. Surgery is very useful for both diagnosis and treatment of a small, solitary, deeply located, pulmonary nodule of IgG4-related lung disease.

## Highlights

• A rare solitary lung nodule of IgG-4 related disease, minicking primary lung carcinoma or metastatic lung tumor, was slowly growing after for colon cancer.
• IgG4-related lung disease is very important to include in the differential diagnosis of a ground glass opacity or a solitary lung nodule.
• Surgery is very useful for both diagnosis and treatment of a solitary lung nodule of IgG-4 related lung disease.

## 1 Introduction

IgG4-related disease, a poorly understood immunomodulated inflammatory disease, characteristically affects various organs, such as the pancreas and parotid glands. The histological findings include IgG4-positive plasma and cell and lymphocyte infiltration and fibrosis. Although lung involvement is rare, it has recently been reported. Pulmonary involvement manifests on chest computed tomography (CT) as a variety of lesions, including mass-like lesions, a bronchovascular nodular pattern, and ground glass opacities. It can therefore mimic other diseases, such as primary lung malignancy, sarcoidosis, and non-specific interstitial pneumonia.

Herein, we present a rare case of a successful surgical diagnosis and treatment of IgG4-related lung disease in a patient with radiological findings of a ground glass opacity that gradually morphed over time into a solid nodule and was suspected of being a primary lung cancer or metastatic tumour. The work has been reported in line with the SCARE criteria [1].

## 2 Presentation of case

**Fig. 1:**
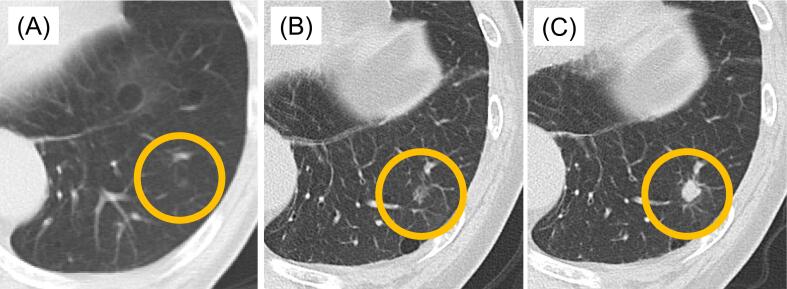
(A) Chest CT images showing a small (4-mm) ground glass opacity. (B—C) The small ground glass opacity was gradually enlarged over two years and six months (B), becoming a small (9-mm) solid nodule with spiculation and lobulation after three years (C).

**Fig. 2:**
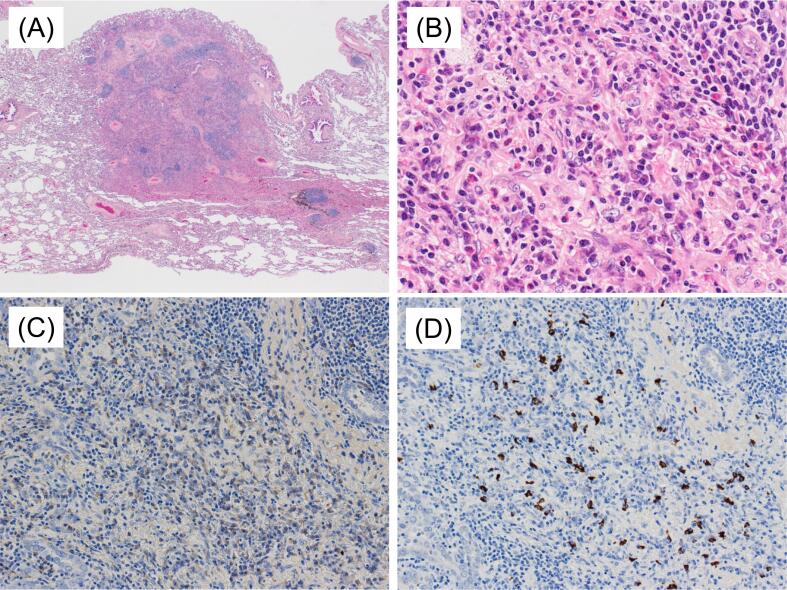
(A-B) Photomicrographs of the pulmonary nodule showing lymphoplasmacytic cell infiltration on hematoxylin and eosin-stainings. Original magnification ×1 (A) and ×40 (B). ()Infiltrates of IgG-positive (C) and IgG4-positive plasma cells (D) are seen on immunohistochemistry-stained sections. Original magnification ×20.

A 76-year-old man was found to have a slowly growing lung nodule on follow-up chest CT after surgery for colon carcinoma. He was a smoker and his medical history included stage 2 sigmoid colon cancer for which he had undergone high anterior resection. Six months after that surgery, follow-up chest CT examination showed a 4-mm ground glass opacity in the left lower lobe of the lung. This lesion gradually became consolidated and enlarged to 9 mm over about three years (Fig. 1). Chest CT examination showed no evidence of mediastinal or hilar lymphadenopathy. The patient's serum carcinoembryonic antigen was 2.1 ng/mL (normal range of 0–5.0 mg/dL). Initially, primary lung adenocarcinoma or metastatic lung tumour were considered the most probable differential diagnoses. Because the lesion was small and deeply imbedded in the lung, it would have been difficult to obtain a biopsy by bronchoscopy for histopathological examination. We therefor performed a video-assisted left basal segmentectomy for the purposes of both diagnosis and treatment. Macroscopically, the tumour was greyish-white. Pathological examination of the operative specimen revealed lymphoplasmacytic infiltration, mainly with IgG4-positive plasma cells, and no evidence of malignancy (Fig. 2). The ration of IgG4-/IgG-positive cells was 80 % and there were 20–30 IgG4-positive cells per high power field. The patient's serum IgG4 concentration was normal at 56 mg/dL (normal range 11–121 mg/dL) after the pulmonary surgery. A probable diagnosis of IgG4-related disease was made in accordance with the comprehensive diagnostic criteria for IgG4-related disease published in 2016 [2]. Our patient was asymptomatic and no other organs were involved; we therefore decided to follow him up without steroid therapy. There has been no evidence of recurrence of IgG4-related lung disease during the 6 months since surgery.

## 3 Discussion

IgG4-related disease, a poorly understood systemic immune system disorder, has specific pathologic, serologic, and clinical features. It consists of tumour-like lesions with lymphoplasmacytic infiltrates and abundant IgG4-positive plasma cells, high IgG4 serum concentrations, and storiform fibrosis. Various organgs, including the pancreas, salivary glands, bile duct, kidney, lung, retroperitoneum, meninges, mediastinum, liver, and thyroid, can also be involved [3].

IgG4-related lung disease was first described in 2004 in a patient with interstitial pneumonia and autoimmune pancreatitis [4]. Pulmonary involvement is usually preceded by involvement of another organ, most commonly the pancreas [5]. The median age of patients at the time of diagnosis of lung nodules is reportedly 60 years, the male: female ratio being 39:11 [3]. A major characteristic of this disease is multiple, small, bilateral, lung nodules and solid nodules reportedly being detected in almost all patients [3]. However, solitary nodules are rare, being present in only 14 % of cases [3]. Reported radiological pulmonary abnormalities include ground-glass opacities, thickening of the pleura, interlobular septa, and bronchial walls, pleural effusion, interstitial changes, and mediastinal or hilar lymphadenopathy [3]. It is difficult to differentiate IgG4-related lung nodules from other lung diseases, such as primary or metastatic lung tumours, standard interstitial pneumonia, organizing pneumonia, non-specific interstitial pneumonia, and sarcoidosis [3]. In our case, the pulmonary lesion initially took the form of a single ground-glass opacity, then gradually became a solid nodule with lobulation and spiculated margins. It wound have been difficult to obtain a biopsy for histopathological examination because the nodule was small and deeply imbedded in the lung. We therefore decided to obtain a biopsy by surgical excision. Inflammatory myofibroblastic tumour or Rosai-Dorfman disease are pathological differential diagnosis. IgG4-related lung disease and Rosai-Dorfan disease may overlap to some extent, but the number of IgG4-positive plasma cells and the IgG4/IgG ratio in IgG4-related lung disease is reported to be higher than in these two diseases. The probable diagnosis was IgG4-related disease according to the comprehensive diagnostic criteria for IgG4-related disease published in 2016 [2]. IgG4 serum concentrations >135–144 mg/dL are reportedly diagnostic, with a sensitivity of 87 % and specificity of 83 % [6]; however, our patient's IgG4 serum concentration was normal.

International consensus was recently reached on management and treatment of IgG4-related disease. According to these criteria, all symptomatic patients and a subset of asymptomatic patients require treatment [5]. Glucocorticoids are the first line agent for active disease unless they are contraindicated [5]. In our case, we decided on observation only in view of the absence of symptoms, normal serum IgG4, and the absence of other lesions after resection.

## 4 Conclusion

We have here presented a rare case of IgG4-related lung disease with a 3-year course, including detailed radiological findings. The solitary lesion was difficult to distinguish from primary lung malignancy or metastatic lung tumour on imaging, particularly because it presented as an isolated, ground glass opacity that gradually morphed into a solid nodule, and no other organs were involved. Surgery was very useful for both diagnosis and treatment.

## Conflict of interest statement

The authors declare that they have no conflicts of interests regarding the publication of this paper.
